# A Stores Superintendent's Views

**Published:** 1920-11-06

**Authors:** 


### THE FOES OF HOSPITAL ECONOMY.

### A Stores Superintendent's Views.

A SUGGESTIVE article in support of co-operation and standardisation in hospital buying lately appeared in the *North Middlesex Chronicle*. The writer was Mr. Sydney Dunstan, Dispensary and General Stores Superintendent at the Royal Victoria Infirmary, Newcastle-on-Tyne. He explained that his argument is based on twenty-five years' experience in the wholesale chemical trade and in hospitals, and that this experience has convinced him that there is a great lack of co-operation, not only between hospitals, but between different departments of the same hospital. The attempts at co-operative buying has in his view hitherto failed to succeed because no authoritative organisation has been placed in control, and only a few big hospitals have joined the scheme, so that the quantities purchased are relatively small. Beside this there are many variations in requirements, so that it is impossible to deal economically with every detail.

Mr. Dunstan complains that in most of the big hospitals the work of the trained pharmacist is usually limited to drugs and dressings. An experienced chemist could superintend the manufacture of "scores of items now bought from the trade," such as custards, disinfectants, essences, jellies, sauces, and inks. Any hospital with a fully-equipped laboratory should use its apparatus, he urges, for the manufacture of all the preparations which it requires. The reason given why this course is not adopted is that enterprise and co-operation are wanting between the various departments. Mr. Dunstan avers that one department is prejudiced against another, and that this prejudice invariably blocks the way. Its removal is the first step towards improvement.

On co-operative buying and its limitations an interesting point is made. Its success would depend upon a central depot, but "many of the larger places in London probably would not benefit to any great extent. But the smaller institutions, especially in the provincial towns, would save large sums of money." Mr. Dunstan foresees few difficulties in the way of such a scheme. If a central depot were started in London it should act not only as a clearing house, but also as a centre of information. It should be able to say what work this hospital or that cost and with what success—an obscure sentence which it would be convenient to have explained. The centre would resemble a wholesale house, the object of which would be not dividends but the assistance of the smaller hospitals. To start the depot certain items of common use in hospitals would be chosen, and a weekly list be issued of those which can be supplied, with their prices. Samples, where necessary, would be submitted, but the option to purchase or not would remain with the institutions. Any hospital able to buy in a better market should give information to the depot, to enable it to benefit its members and increase its usefulness.

Mr. Dunstan illustrates the opportunity of the depot by reference to the enormous variations observed in tenders even for the same article, a variation which may run from 50 to 100 per cent. He explains it because one firm will have to buy in the open market, while another may have large stocks on hand, previously bought at a low figure. The depot should take advantage of these conditions, and thus benefit the smaller institutions. On such a plan, Mr. Dunstan believes, expenditure would be much lessened, materials would be improved, and even work lightened. But these happy results depend entirely, in his opinion, on co-operation and the standardisation of hospitals' supplies.

Brief as the article is, and consequently covering largely familiar ground, it contains more than one interesting suggestion. But, more than this, it is pleasant to see the subject of many private conversations becoming expressed in print. There is much to be said for arguments on paper. Whether or not hospital laboratories attached to the pharmaceutical departments are largely wasted is often casually discussed, but things go on as before, so long as the discussion is carried on in whispers. Again, the inter-departmental jealousy, to which every institution is subject, can never be seen in its true light until instances are produced of its effects upon economy. We should know where we are in this matter, and should like to see Mr. Dunstan return to the subject in greater detail. But he has done a service in interesting the general reader in a side of hospital life which is scarcely ever revealed.

